# Structural and Functional Characterizations of Cancer Targeting Nanoparticles Based on Hepatitis B Virus Capsid

**DOI:** 10.3390/ijms22179140

**Published:** 2021-08-24

**Authors:** Yunseok Heo, Hyeongseop Jeong, Youngki Yoo, Ji-Hye Yun, Bumhan Ryu, Young-je Cha, Bo-Ram Lee, Ye-Eun Jeon, Jongmin Kim, Sojin Jeong, Eunji Jo, Jae-Sung Woo, Jeewon Lee, Hyun-Soo Cho, Weontae Lee

**Affiliations:** 1 Structural Biochemistry & Molecular Biophysics Laboratory, Department of Biochemistry, College of Life Science and Biotechnology, Yonsei University, Seoul 03722, Korea; uoonsek1@yonsei.ac.kr (Y.H.); jihye2@spin.yonsei.ac.kr (J.-H.Y.); yejeon@spin.yonsei.ac.kr (Y.-E.J.); jmkim@spin.yonsei.ac.kr (J.K.); 2 Department of Life Sciences, College of Life Sciences and Biotechnology, Korea University, Seoul 02841, Korea; hsjeong@kbsi.re.kr (H.J.); jaesungwoo@korea.ac.kr (J.-S.W.); 3 Korea Basic Science Institute, Daejeon 28119, Korea; 4 Department of Systems Biology, College of Life Science and Biotechnology, Yonsei University, Seoul 03722, Korea; think8989@gmail.com (Y.Y.); azzogoory@gmail.com (Y.-j.C.); 5 PCG-Biotech, Ltd., Yonsei Engineering Research Park, Yonsei University, Seoul 03722, Korea; 6 Research Solution Center, Institute for Basic Science, Daejeon 34126, Korea; ryubh@ibs.re.kr; 7 Department of Chemical and Biological Engineering, College of Engineering, Korea University, Seoul 02841, Korea; lbr0523@korea.ac.kr (B.-R.L.); amyann109@naver.com (S.J.); ww1291@korea.ac.kr (E.J.)

**Keywords:** cancer therapy, cryo-EM, HBcAg, affibody, EGFR, gold ions

## Abstract

Cancer targeting nanoparticles have been extensively studied, but stable and applicable agents have yet to be developed. Here, we report stable nanoparticles based on hepatitis B core antigen (HBcAg) for cancer therapy. HBcAg monomers assemble into spherical capsids of 180 or 240 subunits. HBcAg was engineered to present an affibody for binding to human epidermal growth factor receptor 1 (EGFR) and to present histidine and tyrosine tags for binding to gold ions. The HBcAg engineered to present affibody and tags (HAF) bound specifically to EGFR and exterminated the EGFR-overexpressing adenocarcinomas under alternating magnetic field (AMF) after binding with gold ions. Using cryogenic electron microscopy (cryo-EM), we obtained the molecular structures of recombinant HAF and found that the overall structure of HAF was the same as that of HBcAg, except with the affibody on the spike. Therefore, HAF is viable for cancer therapy with the advantage of maintaining a stable capsid form. If the affibody in HAF is replaced with a specific sequence to bind to another targetable disease protein, the nanoparticles can be used for drug development over a wide spectrum.

## 1. Introduction

If a multifunctional agent that can perform delivery to the disease site and provide therapeutic function is developed, it will play a significant role in the treatment of cancer. Recently, researchers have attempted to develop such agents [1,2,3,4,5]. However, in many cases, such agents had the drawbacks of cytotoxicity and/or instability. To overcome these negative effects, we developed nanoparticles based on hepatitis B core antigen (HBcAg). The nanoparticles bound specifically to a tumor cell receptor, performed magnetic hyperthermia-based ablation of tumor cells, and were then spontaneously disassembled over time—exhibiting negligible cytotoxicity. However, the binding mechanism between the nanoparticles and tumor cell receptor could not be fully explained without 3D structures of the nanoparticles. Therefore, we obtained the molecular structures of the nanoparticles using cryogenic electron microscopy (cryo-EM).

HBcAg monomers form a dimer and spontaneously assemble into capsids composed of 180 (T = 3 state), or 240 (T = 4 state) subunits [6,7]. Therefore, the nanoparticles based on HBcAg have molecular weight (MW) of over 6000 kDa. The nanoparticles adopted a tandem repeat of affibody for binding to the tumor receptor, human epidermal growth factor receptor 1 (EGFR). The scaffold of the original affibody is the immunoglobulin G (IgG) binding domain. The affibody was modified to bind with high affinity to specific proteins. The affibody used in this study specifically binds to the extracellular domain of EGFR with a dissociation constant (K_D_) of 5.4 nM [8]. EGFR is a transmembrane receptor for specific ligands. EGFR is activated by an epidermal growth factor, and then stimulates signal transduction cascades leading to DNA synthesis and cell proliferation [9]. Therefore, mutations that cause overexpression of EGFR are associated with a number of cancers [10].

Cryo-EM uses samples cooled to cryogenic temperatures. The development of advanced detector and software algorithms enabled the determination of protein structures at a near-atomic resolution [11]. Unlike NMR spectroscopy, which has a size limitation for structural analysis, or X-ray crystallography, which requires crystallization, cryo-EM can be used to determine macromolecular structures without crystallization. We obtained the molecular structures of HBcAg engineered to present affibody and tags (HAF) using cryo-EM. The structure of HAF complexed with EGFR (HAFE) was also analyzed from the 3D reconstruction structures using cryo-EM.

The development of agents that can be applied to cancer therapy is the next step for cancer treatment. We inspected clinically promising nanoparticles for cancer therapy. In vitro binding of recombinant nanoparticles (HAF) to the recombinant extracellular domain of EGFR was confirmed. The adenocarcinoma cells which overexpressed EGFR were used to verify the therapeutic effect of HAF complexed with gold ions (HAFG). HAFG destroyed adenocarcinoma cells through magnetic hyperthermia therapy induced by an alternating magnetic field (AMF). The molecular structures of HBcAg were previously reported by either X-ray crystallography or cryo-EM [12,13,14,15,16,17,18]. However, this is the first report of a modified HBcAg structure for cancer-targeting. Based on our structure, another nanoparticle can be designed for a different purpose by changing the sequence of the affibody, or by replacing the affibody with another protein. Our modified HBcAg structure provides insight into the ability to manipulate HBcAg for a desired purpose, and our results promote the development of another multifunctional agent for cancer therapy.

## 2. Results

### 2.1. Sequential and Structural Differences between HBcAg and HAF

**Figure 1:**
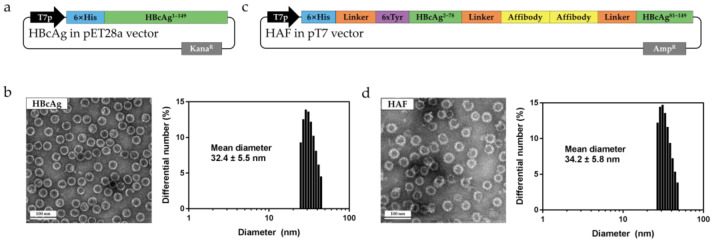
The recombinant plasmid map, TEM, and DLS of HBcAg and HAF. (**a**) Schematic plasmid vector used for expressing HBcAg. The construct is composed of the 6× histidine tag followed by the N-terminal domain of HBcAg (aa 1–149). (**b**) TEM and DLS analysis of purified HBcAg. The measured diameters of HBcAg from the TEM and DLS matched each other. (**c**) Schematic plasmid vector used for expressing HAF. The HBcAg (aa 1–149) was divided into two parts. The affibody was inserted for binding to EGFR. The 6× histidine and 6× tyrosine tags were used for binding to the gold ions. (**d**) TEM and DLS analysis of purified HAF. The mean diameters of HAF from the TEM and DLS were in agreement.

HBcAg consists of two domains: the N-terminal domain (aa 1–149) for assembly and the C-terminal domain (aa 150–183) for binding to nucleic acids [19]. The N-terminal domain of HBcAg (aa 1–149) can form a dimer and be assembled into a capsid with an icosahedral symmetry composed of 180 (T = 3 state) or 240 (T = 4 state) subunits. Accordingly, the N-terminal domain is typically chosen for use in structural studies, as it was in this study [12,13,14,15,20,21,22,23,24,25]. HBcAg was expressed in *Escherichia coli* (*E. coli*) using the pET28a vector (Figure 1a) and purified using sucrose gradient centrifugation and size exclusion chromatography (SEC) (Supplementary Figure S1a,b). HBcAg was self-assembled into the T = 4 and T = 3 symmetry capsids. HBcAg was visualized through transmission electron microscopy (TEM) and the diameter of the HBcAg was found to be 31–35 nm (Figure 1b). We also used dynamic light scattering (DLS) to measure the diameter of the HBcAg particles. The measured mean diameter of HBcAg was 32.4 nm, which was in agreement with the diameter measured using TEM (Figure 1b). The purified HBcAg favored the T = 4 state, as previously reported [17,18]. However, the population difference between T = 4 and T = 3 of the purified HBcAg was not as large as that reported in previous papers. The 6× histidine tag may have affected the population (Figure 1a).

HAF is composed of HBcAg, along with histidine and tyrosine tags for binding to gold ions, as well as a tandem repeat of the affibody for binding to EGFR (Figure 1c). The linkers (spacer peptides) were inserted between each component (Figure 1c). The gold ions that were bound to HAF induced a hyperthermia effect under the magnetic field. HAF was designed to present the affibody on the spike, which is based on the HBcAg molecular structure. In the HAF sequence, the affibody was inserted between α4 and α5 of the HBcAg, the spike tip of the HBcAg (Supplementary Figure S2). We deliberately designed the affibody to appear at the outermost edge of the spike. This is because the affibody on the spike of HAF will facilitate the binding to EGFR by minimizing collisions in terms of the space-filling model. HAF was expressed in *E. coli* and self-assembled into a spherical capsid form. The assembled HAF was collected by sucrose gradient centrifugation, and further purified by SEC (Supplementary Figure S3a,b). Because the MW of HAF is high due to the assembled capsid form, HAF was eluted in void volume (Supplementary Figure S3b). The fractions of the SEC peak were visualized using sodium dodecyl sulfate polyacrylamide gel electrophoresis (SDS-PAGE). In the gel, the band appeared at 37 kDa, the monomer size of HAF (Supplementary Figure S3b). As a result of SEC purification, it was found that the only capsid form of HAF existed at the void volume peak. The purified HAF preferred the T = 4 symmetry. The diameter of HAF was slightly larger than that of HBcAg, which is ascribed to the affibody. The mean diameter of HAF was found to be 35.0 nm, as measured by TEM, and 34.2 nm when measured by DLS (Figure 1d). From the above results, it was found that HAF assembled into a capsid similar to that of HBcAg, despite the considerable sequential modification.

### 2.2. Thermostability of HAF

**Figure 2:**
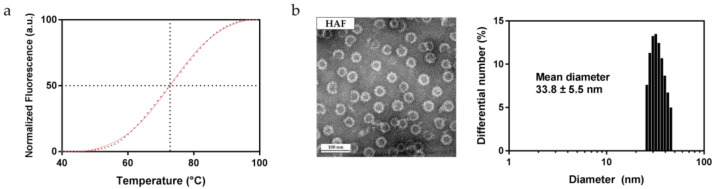
Thermostability of HAF. (**a**) Melting temperature analysis of HAF. The thiol-reactive probe was used for plotting the melting temperature curve of HAF. The calculated melting temperature of HAF was 72.80 °C. (**b**) TEM and DLS analysis of HAF after 7 days incubated at 42 °C. The results from the TEM and DLS suggest that HAF can maintain a capsid structure for at least 7 days at 42 °C.

HAF was designed to be delivered and bound to subcutaneous tumors through the blood vessels. Testing the thermostability of HAF is essential as it can be denatured during delivery if it is not heat resistant. Melting temperature (Tm) is one of the indicators that can be used to check the thermal stability of proteins. We used a thiol-reactive probe BODIPY FL L-cystine (BFC) to measure the Tm of HAF. The BFC probe is a reporter dye and the fluorescent signal can be detected with a standard real-time polymerase chain reaction (RT-PCR) machine. The probe was validated for Tm measurements of several proteins, and showed sufficient stability when compared with conventional reagents [26]. The change of fluorescence caused by the protein unfolding was plotted against temperature [27]. The measured Tm of HAF was 72.80 °C (Figure 2a). The fluorescence values were normalized from 0 to 100, and since temperatures from 4 °C to 40 °C showed no change in fluorescence values they were omitted from the figure. Each point was connected through sigmoidal fitting.

HBcAg, the scaffold of HAF, has a Tm of 70 °C [28]. HAF, which has a tandem repeat of affibody, is more stable to heat than HBcAg as based on Tm values. The unusual Tm values of HAF and HBcAg are attributed to the massive and stable capsid structure with symmetry. We performed tests to determine if the capsid structure of HAF would be maintained during delivery. HAF was incubated at 42 °C for 7 days before TEM and DLS analysis. The TEM images of HAF showed that HAF still held the ordered capsid structure (Figure 2b) and that the diameter was 33–37 nm. DLS also verified that HAF incubated at 42 °C had almost the same diameter as that of the general HAF (Figure 2b). The results suggest that HAF would not be disassembled due to thermal factors even after inhabiting the blood vessels for 7 days.

### 2.3. Cryo-EM Structures of HAF

The structures of HBcAg were previously elucidated using X-ray crystallography and cryo-EM. The structures of HBcAg complexed with various compounds have also been identified [15,17]; however, the structures of nanoparticles based on HBcAg and modified for use in cancer therapy have not yet been determined. The HAF used in this study was made by inserting the affibody into the outermost edge of the spike. We measured the approximate size and shape of HAF using TEM and DLS; however, it was necessary to obtain the 3D structure of HAF using cryo-EM to compare HAF and HBcAg more accurately.

**Figure 3:**
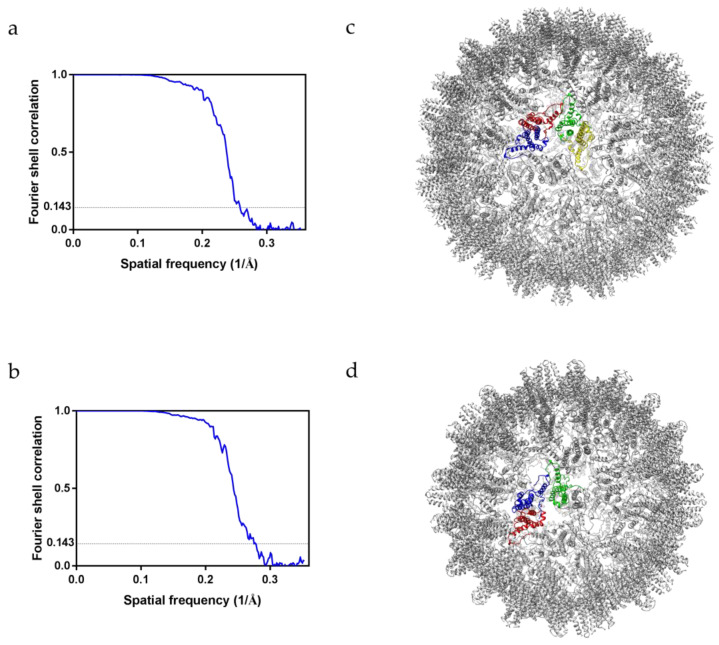
FSC curves and molecular structures of HAF. (**a**) FSC curve of HAF (T = 4 state) reconstruction. The dashed line indicates the correlation of 0.143 which was used as cutoff for determining the resolution. The final resolution is 3.86 Å. (**b**) FSC curve of HAF (T = 3 state) reconstruction. The dashed line indicates the correlation of 0.143 which was used as cutoff for determining the resolution. The final resolution is 3.60 Å. (**c**) The molecular structure of HAF (T = 4 state). The four subunits forming the asymmetric unit are colored in red, blue, green, and yellow. (**d**) The molecular structure of HAF (T = 3 state). The three subunits forming the asymmetric unit are colored in red, blue, and green.

Purified HAF was concentrated to 10 mg/mL to obtain the 3D structure using cryo-EM. The HAF sample was loaded onto positively glow-discharged grids. The grids were blotted for 6 s and plunged into liquid ethane cooled by liquid nitrogen. The cryo-EM micrographs of HAF were collected using a Titan Krios 300 kV transmission electron microscope and processed using cryoSPARC [29]. In the micrograph, the HAF particles were well dispersed and the size of HAF was similar to that recorded in the TEM results (Supplementary Figure S4a). Suitable micrographs were selected and 2D class-averaged images were obtained using the HAF particles in the micrographs (Supplementary Figure S4b,c). The 2D class-averaged images were used for the 3D reconstruction of HAF. A Fourier shell correlation (FSC) of 0.143 was chosen as the cutoff for resolution determination, and the final resolutions of HAF (T = 4 state) and HAF (T = 3 state) were 3.86 Å and 3.60 Å, respectively (Figure 3a,b). The density map of the affibody was not clearly visible since the connected linkers induced flexibility of the affibody, causing the structures of HAF to appear very similar to those of the HBcAg (Supplementary Figure S4d,e). The four subunits forming the asymmetric unit of HAF (T = 4 state) are colored in red, blue, green, and yellow (Figure 3c). The three subunits forming the asymmetric unit of HAF (T = 3 state) are colored in red, blue, and green (Figure 3d). The data acquisition conditions and the model validation are summarized in Table 1.

As a result, it was confirmed that HAF, in which the affibody was inserted by manipulating the spike tip, has a similar overall structure to that of HBcAg. Because HAF stably maintains the capsid form, HAF is a suitable nanoparticle for stably targeting and treating cancers that overexpress EGFR.

### 2.4. In Vitro Binding of HAF and EGFR

**Figure 4:**
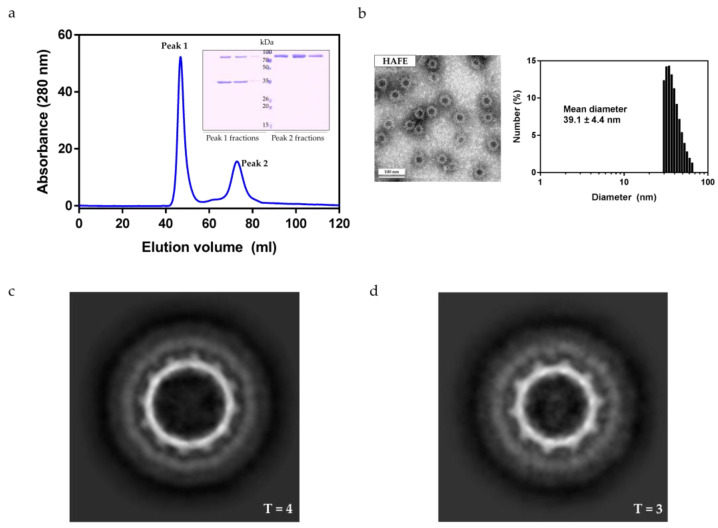
SEC of HAFE and structural analysis of HAFE. (**a**) SEC and SDS-PAGE analysis of HAFE. The purified HAF and EGFR mixture (molar ratio 1:2) were injected into an SEC column. Peak 1 and Peak 2 fractions were visualized through SDS-PAGE analysis. HAFE was eluted in void volume (Peak 1), and the residual EGFR was eluted in Peak 2. (**b**) TEM and DLS analysis of purified HAFE. The measured mean diameters of HAFE were longer than those of HAF, which was attributed to the EGFR being bound to HAF. (**c**) The most populated 2D class-averaged images of HAFE (T = 4 state). (**d**) The most populated 2D class-averaged images of HAFE (T = 3 state).

The extracellular domain of EGFR was expressed in *Spodoptera frugiperda* (Sf9) insect cells in secreted form. EGFR was purified using an NHS-activated HP column and SEC, as described previously [30]. The affibody of HAF binds to the extracellular domain of EGFR with a K_D_ of 5.4 nM [8], and we attempted to confirm this binding using SEC. The purified HAF and EGFR were mixed at a molar ratio of 1:2, incubated at 4 °C for 1 h, and then injected into an SEC column. When the SEC peaks were analyzed using SDS-PAGE, it was found that HAF and EGFR appeared together in Peak 1, and the residual EGFR was eluted in Peak 2 (Figure 4a). The extracellular domain of EGFR seemed to bind to HAF as HAF and EGFR were eluted together in the void volume (Peak 1). To verify that the EGFR bound to HAF, the fractions in which the HAF and EGFR were eluted together were analyzed by TEM and DLS. The diameters measured from the TEM images were 36–43 nm (Figure 4b). The mean diameter from the DLS was 39.1 nm (Figure 4b). Since the mean diameter of the particle was significantly larger than that of HAF, we were able to confirm that these particles were HAF complexed with EGFR (HAFE).

HAFE was also visualized using cryo-EM. The purified HAFE was concentrated to 12 mg/mL. The cryo-EM data collection was carried out similarly to that of HAF. The data acquisition conditions are summarized in Table 1. In the cryo-EM micrographs, it was confirmed that EGFR was attached to the outside of HAF, which was clearly different from the micrograph of HAF. The HAFE particles in the cryo-EM micrographs were collected and averaged. Both forms (T = 4 and T = 3) of HAFE were obtained through 2D classification (Figure 4c,d). In the 2D class-averaged images of HAFE, it was found that the EGFR was bluish on the outside of the HAF (Figure 4c,d). Therefore, it is assumed that EGFR binds to HAF by surrounding it on the outside.

### 2.5. Confirmation of the Affibody Conformation Using Cryo-EM

The conformation of affibody could not be confirmed from the cryo-EM structures of HAF. The main reason for this problem is the linkers with long length before and after each affibody (Supplementary Figure S2). Each linker composed of 10 or more residues made the affibody flexible, and thus the structure of the affibody in HAF could not be clearly confirmed even though the molecular structure of HAF was obtained. In order to solve this problem, we designed new HAF constructs with short linkers. However, if the length of the linkers is too short, the construct may not form a capsid, so constructs with linkers of various lengths were prepared. Even if each linker was composed of two residues, it was confirmed that the construct could assemble into capsids. The sequences of HAF with short linkers (HAFS) and HAF were aligned (Supplementary Figure S5).

HAFS was self-assembled into a spherical capsid form, and the assembled HAFS was collected using sucrose gradient centrifugation. The fractions with higher sucrose concentration were used for further purification using SEC. The fractions of the void volume peak of SEC were concentrated to prepare the cryo-EM sample. The cryo-EM data of HAFS were collected in a similar routine to HAF. The number of HAFS particles per micrograph was sufficient (Supplementary Figure S6a). The micrographs were processed using cryoSPARC [29]. The sorted micrographs were used for 2D classification. In the 2D class-averaged image of HAFS, the affibody density was clearly identified (Supplementary Figure S6b). On the other hand, the affibody density could not be confirmed in the 2D class-averaged image of HAF (Supplementary Figure S4b,c). The 3D reconstruction structure of HAFS was also obtained, and the final resolution of HAFS (T = 4 state) was 4.41 Å (Supplementary Figure S6c). Detailed information for the data collection and model validation is summarized in Table 1.

**Figure 5:**
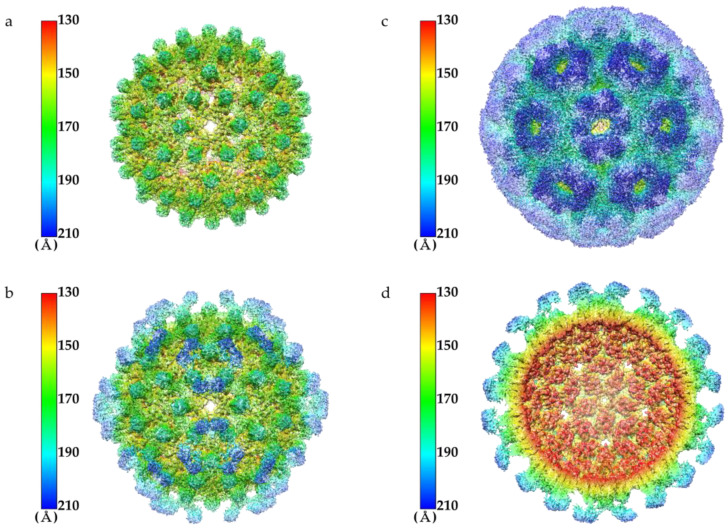
The cryo-EM structures of HAFS (T = 4 state). (**a**) The density map of HAFS was colored by radius and contoured at the threshold value of 0.44 to illustrate the capsid density. (**b**) The density map of HAFS was contoured at the threshold value of 0.33 to illustrate the affibody density. Part of the affibody density is visible. (**c**) The density map of HAFS was contoured at the threshold value of 0.11 to highlight the affibody density. The affibody density is fully visible. (**d**) The density map of HAFS was cut and contoured at the threshold value of 0.33 to illustrate the inner part and cross-section of the affibody. The density map and molecular structure of HAFS were superimposed in this figure.

In the cryo-EM structure of HAFS, the capsid part of HAFS had the same size and shape as that of HAF or HBcAg (Figure 5a). When the threshold was lowered, the affibody density protruding on both sides of the HAFS spike was clearly visible (Figure 5b). Each spike is composed of HAFS dimer, and the affibody density protruding from the HAFS monomer consists of a pair of affibodies coming out of and back into the spike. In addition, at this threshold, only the affibody of HAFS, which consists of three protrusions in the three-fold axis, was observed. Lowering the threshold further revealed all affibodies (Figure 5c). The full affibody density perfectly surrounds the HAFS. The density map of HAFS was cut to represent the inner part (Figure 5d). As a result, the affibody density extending from one spike to the left and right is more clearly visible. From the above results, it was confirmed that HAFS formed a spherical capsid and the affibody density was much more clearly visible than in the case of HAF. Therefore, we obtained detailed conformational information of the affibody in HAFS. Based on HAFS, the affibody part can be replaced with different protein sequences associated with human diseases to create new nanoparticles with different purposes.

### 2.6. Therapeutic Effect of HAFG on EGFR Overexpressing Adenocarcinoma

EGFR-overexpressing HT-29 cells and A431 cells were cultured in 96-well plates. Both cells were treated with rabbit anti-EGFR IgG, followed by incubation with fluorescein isothiocyanate (FITC)-conjugated mouse anti-rabbit secondary antibodies for 45 min at room temperature. EGFR was visualized as a green color by FITC, and 4′,6-diamidino-2-phenylindole (DAPI) was used to stain the nuclei of adenocarcinoma cells in blue. In the merged image, it was confirmed that both cell lines overexpress EGFR (Supplementary Figure S7). As HAFG binds specifically to EGFR, Cy5.5-labeled HAFG would be used to diagnose EGFR-overexpressing cancers by monitoring the fluorescence.

**Figure 6:**
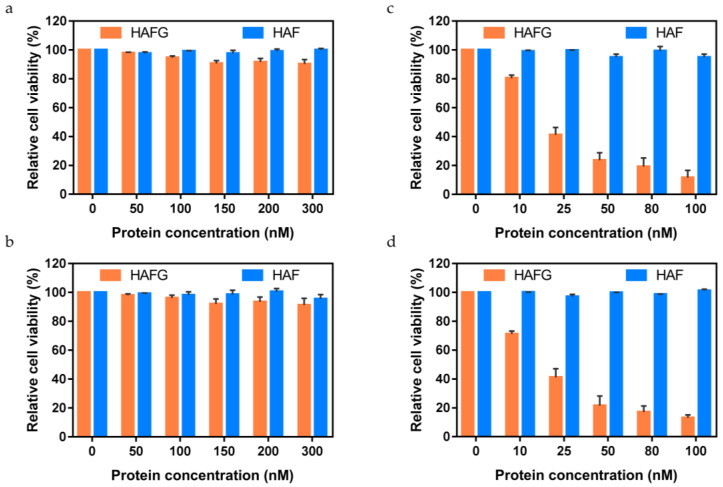
CCK-8 assay results to estimate the therapeutic effect of HAFG. (**a**) CCK-8 assay result of EGFR-overexpressing HT-29 cells. The cell viability was constant regardless of the concentration of HAF or HAFG. (**b**) CCK-8 assay result of EGFR-overexpressing A431 cells. The cell viability was constant regardless of the concentration of HAF or HAFG. (**c**) CCK-8 assay result of EGFR-overexpressing HT-29 cells under AMF. The cell viability decreased remarkably in proportion to the concentration of HAFG. (**d**) CCK-8 assay result of EGFR-overexpressing A431 cells under AMF. The cell viability decreased remarkably in proportion to the concentration of HAFG. In this figure, mean and standard deviation are presented (*n* = 6). The viability measured without protein was presented as 100% for each graph.

Nanoparticles bound to metal ions are toxic when accumulated [31,32,33]. Synthetic nanoparticles over 8 nm cannot undergo renal clearance through glomerulus filtration and accumulate in organs, causing toxicity such as necrosis and nephritis [34,35]. However, in contrast to synthetic nanoparticles that accumulate in vivo without degradation, self-assembled HAFG spontaneously disassembles into individual subunits over time, which can then be degraded and released through glomerular filtration. An absence of cytotoxicity of nanoparticles, as well as their therapeutic effect, are factors that should be considered prior to selecting nanoparticles as a feasible agent. The therapeutic function and cytotoxicity of HAFG could be estimated by measuring the relative cell viability. The cell counting kit (CCK-8) assay was used to estimate this viability. The CCK-8 assay measures the mitochondrial reductase activity in live cells. The results demonstrated that, regardless of the concentration of HAF or HAFG in HT-29 cells, the cell viability was similar to that of the negative control (free HT-29 cells) (Figure 6a). In the case of A431, cell viability was also constant regardless of the concentration of HAF or HAFG (Figure 6b). This indicates that both HAF and HAFG have negligible cytotoxicity. There was no cytotoxicity, even when using human lung fibroblast cells (WI-38) rather than the abovementioned adenocarcinoma cells (Supplementary Figure S8). Therefore, HAFG is a biocompatible nanoparticle because it is not cytotoxic by itself and does not accumulate as it can be automatically degraded.

After AMF with a frequency of 360 kHz and amplitude of 10 kW was applied to the HT-29 cells for 30 min, the cell viability decreased remarkably in proportion to the concentration of HAFG (Figure 6c). As AMF was applied for 30 min to the A431 cells, the cell viability was also reduced significantly according to the concentration of HAFG (Figure 6d). In summary, HAFG itself was not cytotoxic (Figure 6a,b), and HAFG killed the EGFR-overexpressing tumor cells via the magnetic hyperthermia effect under AMF (Figure 6c,d). The fact that HAFG can bind specifically to tumors, destroy the tumors without direct resection, and be degraded automatically over time suggests that HAFG is a clinically suitable candidate for a cancer therapy agent.

## 3. Discussion

Nanoparticles that can viably be used for targeting and ablating cancer were verified. The nanoparticles (referred to as HAF in this paper) were expressed in *E. coli* and purified for functional and structural studies. HAF binds to the recombinant extracellular domain of EGFR which is overexpressed in various cancers. HAF complexed with gold ions (HAFG) showed negligible cytotoxicity, and destroyed EGFR-overexpressing adenocarcinoma cells via the hyperthermia effect under AMF. Cryo-EM was used to elucidate the 3D structures of HAF. The overall structure of HAF was not significantly different from that of HBcAg, except in having the affibody on the spike. As the affibody had a flexible connection, a solid density map of the affibody could not be obtained. The optimized construct with shorter linker length (HAFS) was prepared to confirm the affibody density. The cryo-EM structure of the new construct revealed that the affibody stretched out of the spike. Compared with the published structures of HBcAg, the site of the manipulation did not affect capsid formation. Accordingly, our HAF and HAFS structures will be helpful for the development of nanoparticles that have HBcAg as a scaffold. If the affibody of HAF is replaced with another protein sequence, the new nanoparticle so produced can perform the desired function by binding to a completely different target. A more advanced form for cancer therapy could potentially be developed based on our structure.

The stability of nanoparticles is very important as they were designed to be transmitted to the tumor site through the blood vessels. HAFG has HBcAg as a scaffold, meaning that HAFG is also stable to heat. It was confirmed that HAFG would maintain the capsid conformation during delivery to the tumor site through blood vessels. Biodegradability is another important factor for nanoparticles. Many synthetic nanoparticles previously developed are sufficiently stable, but have caused an accumulation of metal ions in vivo since they are not degraded and/or excreted, which can lead to severe negative effects. HAFG, however, is disassembled into individual subunits over time and can be degraded automatically. Accordingly, HAFG achieved stability and biodegradability, as well as the ability to bind strongly and specifically to the extracellular domain of EGFR. The therapeutic function of HAFG was confirmed by measuring the cell viability of adenocarcinoma cells after binding to HAFG under AMF. Based on the above results, HAFG overcame the shortcoming of previously studied nanoparticles and performed the function of a cancer therapy agent.

In conclusion, HAFG performed remarkable multimodal functions as a cancer therapy agent, while overcoming all known shortcomings of other nanoparticles. It is expected that the stable and effective HAFG will soon become a promising and clinically feasible candidate for cancer therapy.

## 4. Materials and Methods

### 4.1. Protein Expression and Purification

HBcAg was expressed and purified following previously described methods [36]. Briefly, HBcAg was expressed in *E. coli* BL21 (DE3) cells (Dynebio, Gyeonggi-do, Korea). Cells expressing HBcAg were grown in Luria–Bertani (LB) media at 37 °C to an optical density at 600 nm (OD_600_) of ~0.6. Protein expression was induced with 1 mM isopropyl-β-D-thiogalactopyranoside (IPTG, LPS Solution, Daejeon, Korea) at 18 °C. The cells were harvested by centrifugation after 16 h of induction and then stored at −70 °C. The cell pellets were resuspended in lysis buffer containing a protease inhibitor cocktail (Roche, Basel, Switzerland), and disrupted by sonication. The lysate was then centrifuged at 4 °C for 30 min. The supernatants were purified using ammonium sulfate precipitation. The precipitants were subjected to sucrose gradient centrifugation. The fractions containing the capsid form of HBcAg were loaded onto a Superdex 200 16/60 column (GE Healthcare, Chicago, IL, USA).

HAF was also expressed and purified similarly to HBcAg. Briefly, HAF was expressed in *E. coli* BL21 (DE3) cells. Cells expressing HAF were grown in LB media at 37 °C to an optical density at 600 nm (OD_600_) of ~0.6. Protein expression was induced with 1 mM IPTG at 18 °C. The cells were harvested by centrifugation after 16 h of induction and then stored at −70 °C. The cell pellets were resuspended in lysis buffer containing protease inhibitor cocktail, and disrupted by sonication. The lysate was then centrifuged at 4 °C for 30 min. The supernatants were purified using ammonium sulfate precipitation. The precipitants were subjected to sucrose gradient centrifugation. The fractions containing the capsid form of HAF were loaded onto a Superdex 200 16/60 column. For the preparation of HAFG, previously described methods for the synthesis of gold nanoparticles were used [37]. One milligram of (CH_3_)_3_PAuCl (Sigma-Aldrich, St. Louis, MO, USA) was added to 1 mg of HAF in 0.2 mL. After stirring for 6 h at room temperature, the mixture was centrifuged for 3 min at 5000 rpm. The supernatant was reacted with 0.02 mL of 500 mM NaBH_4_ (Sigma-Aldrich, St. Louis, MO, USA) for 5 min at room temperature. After the buffer of HAFG was exchanged for phosphate buffered saline (PBS, pH 7.4), the HAFG was used for the CCK-8 assay.

The extracellular domain of EGFR was expressed and purified as described previously [30]. Briefly, the EGFR gene was subcloned into a baculovirus expression vector with an EGFR signal peptide sequence. The expression of the extracellular domain of EGFR in insect cells was carried out using a Bac-to-Bac Baculovirus Expression System (Invitrogen, Waltham, MA, USA). The resulting construct was expressed in Sf9 insect cells in a secreted form. The media containing secreted EGFR were collected through centrifugation to remove the Sf9 cells, and purified using an NHS-activated HP column (GE Healthcare, Chicago, IL, USA) coupled with ER2-IgG proteins (anti-EGFR antibodies). The eluted EGFR was loaded onto a Superdex 200 26/60 column (GE Healthcare, Chicago, IL, USA).

### 4.2. Transmission Electron Microscopy and Dynamic Light Scattering

Recombinant proteins were visualized using a TEM (CM-200 Electron Microscope, Philips, CA, USA) operating at an acceleration voltage of 80 kV. For the preparation of TEM samples, one drop of each capsid particle suspension was placed onto a 200-mesh copper grid precoated with carbon. After 2 min of deposition, distilled water was removed by air-drying. Negative staining was applied using a droplet of 2% (*w/v*) aqueous uranyl acetate solution. The particle size of the recombinant proteins was measured using DLS (NanoBrook 90Plus, Brookhaven, NY, USA).

### 4.3. Melting Temperature Measurement

A thiol-reactive probe was used to detect the fluorescent signal using a real-time polymerase chain reaction machine (CFX96, Bio-Rad, Hercules, CA, USA). The run was initially set to 4 °C, and then increased by 1 °C per min to 99 °C. The data were analyzed by normalizing the fluorescence intensities.

### 4.4. Cryo-EM Data Collection and Processing

Four microliters of HAF samples were applied to positively glow-discharged carbon grids (Quantifoil R1.2/1.3 Cu 200 Mesh, SPI Supplies, West Chester, PA, USA). The grids were blotted for 6 s using Vitrobot Mark IV (Thermo Fisher Scientific, Waltham, MA, USA) at 4 °C with 95% humidity, and plunged into liquid ethane cooled by liquid nitrogen. The cryo-EM images of the HAF particles were collected at the Korea Basic Science Institute using a Titan Krios (Thermo Fisher Scientific, Waltham, MA, USA) transmission electron microscope operated at 300 kV. Movie data were recorded using a Falcon 3EC direct electron detector (Thermo Fisher Scientific, Waltham, MA, USA) using electron counting mode and automatic data acquisition software (EPU, Thermo Fisher Scientific, Waltham, MA, USA). Cryo-EM images of the HAF were processed using cryoSPARC [29]. Micrographs deemed unsuitable for image processing by manual inspection were removed. Then, 308,723 particle images were picked from 1450 micrographs and extracted into 350-pixel boxes. Eight rounds of 2D classifications were carried out to eliminate poorly aligned particles. The number of particles selected for further processing (T = 4 and T = 3) were 68,577 and 50,688, respectively. Subsequently, 3D initial models from 2D particle images were generated. After 3D refinement, the estimated resolution of the final map was 3.86 Å (for T = 4 state) and 3.60 Å (for T = 3 state) based on the 0.143 FSC criterion. The cryo-EM data of HAFS were collected and processed using a similar routine. The number of particles selected for 3D reconstruction (T = 4) was 46,346. The cryo-EM data of HAFE were collected using a similar routine. The number of particles selected for 2D classification (T = 4 and T = 3) were 15,757 and 8753, respectively.

### 4.5. Model Building and Refinement

The cryo-EM structure of the HBcAg (PDB ID: 6BVF) was rigid-body fitted into the HAF density maps using UCSF Chimera and refined using “PHENIX real space refine” in the PHENIX software suite [38]. The geometric restraints for HAF were optimized using the eLBOW module in PHENIX. The quality of the final models was evaluated using the “comprehensive model validation” section and MolProbity in PHENIX. The HAFS was built and refined using a similar routine. Detailed statistics for refinement and validation are included in Table 1. Structures were visualized and figures were produced using the UCSF Chimera and PyMOL programs.

### 4.6. Cell Count Kit

The cell viability of adenocarcinoma was estimated using the CCK-8 assay (Dojindo Molecular Technology, Rockville, MD, USA). HT-29 and A431 cells were cultured in 96-well plates at a density of 1 × 10^4^ cells per well for 24 h. HAF or HAFG at various concentrations were added to the cell cultures in the plates. After 24 h incubation, cells were incubated with CCK-8 reagent for 1.5 h for the CCK-8 assay and the plates were read immediately in a microplate reader at 450 nm. AMF with a frequency of 360 kHz and amplitude of 10 kW was optionally applied for 30 min prior to CCK-8 reagent incubation. The assay results are presented as means ± standard deviation (*n* = 6). The CCK-8 assay results are presented considering the absorbance at 450 nm of non-treated cells as 100%. The cell viability of human lung fibroblast cells (WI-38) was also estimated with a similar routine.

## Figures and Tables

**Table 1:** Summary of data collection, structure determination, and model building of HAF, HAFE, and HAFS.

**Cryo-EM Data Collection and Image Processing**				
Sample	HAF (T = 3)	HAF (T = 4)	HAFE (T = 3 and T = 4)	HAFS (T = 4)
Microscope	Titan Krios	Titan Krios	Titan Krios	Titan Krios
Camera	Falcon 3EC	Falcon 3EC	Falcon 3EC	Falcon 3EC
Voltage (kV)	300	300	300	300
Electron exposure (e-/Å2)	51	51	51	45
Defocus range (µm)	−1.50 ~ −2.50	−1.50 ~ −2.50	−1.50 ~ −2.50	−0.75 ~ −2.75
Pixel size (Å)	1.4	1.4	1.4	1.4
Software	cryoSPARC	cryoSPARC	cryoSPARC	cryoSPARC
Symmetry imposed	I	I	I	I
Overall map resolution (Å)	3.60	3.86	-	4.41
**Atomic Model Refinement (Asymmetric Unit)**				
Software	Phenix	Phenix		Phenix
T number	3	4		4
Model composition				
Chains	3	4		4
Non-hydrogen atoms	3,402	4,536		4,416
Protein residues	423	564		548
Resolution Estimates (Å)				
d FSC model (0/0.143/0.5)	3.4/3.6/4.0	3.6/3.8/4.1		4.2/4.3/4.8
B factor (min/max/mean)	59.71/201.89/101.89	52.69/227.81/104.53		90.35/377.36/159.85
R.m.s. deviations				
Bond lengths (Å)	0.003	0.003		0.003
Bond angles (°)	0.664	0.638		0.622
MolProbity score	1.55	1.50		1.86
Clash score	6.39	5.80		10.29
Rotamer outliers (%)	0.00	0.00		0.00
Ramachandran plot				
Favored (%)	96.84	96.90		95.30
Allowed (%)	3.16	3.10		4.70
Outliers (%)	0.00	0.00		0.00

## Data Availability

The cryo-EM electron density maps for HAF (T = 3), HAF (T = 4), and HAFS (T = 4) have been deposited in the EM Data Bank under the accession codes EMD-31233, EMD-31234, and EMD-31545, respectively. The atomic coordinates for HAF (T = 3), HAF (T = 4), and HAFS (T = 4) have been deposited in the Protein Data Bank under the accession codes 7EOY, 7EP6, and 7FDJ, respectively.

## Supplementary Materials

The following are available online at https://www.mdpi.com/article/10.3390/ijms22179140/s1.
