# Supplementary material for: Structural and Functional Characterizations of Cancer Targeting Nanoparticles Based on Hepatitis B Virus Capsid
Source: Int J Mol Sci. 2021 Aug 24;22(17):9140. doi: 10.3390/ijms22179140 (PMC8430771; doi:10.3390/ijms22179140)
Supplement: Supplementary file 1 [file ijms-22-09140-s001.zip › ijms-1325116-supplementary.pdf]

International Journal of *Molecular Sciences*

MDPI

Structural and Functional Characterizations of Cancer Targeting Nanoparticles Based on Hepatitis B Virus Capsid

Yunseok Heo [1,†], Hyeongseop Jeong [2,3,†], Youngki Yoo [4], Ji-Hye Yun [1,5], Bumhan Ryu [6], Young-je Cha [4], Bo-Ram Lee [7], Ye-Eun Jeon [1], Jongmin Kim [1], Sojin Jeong [7], Eunji Jo [7], Jae-Sung Woo [2], Jeewon Lee [7,*], Hyun-Soo Cho [4,*] and Weontae Lee [1,5,*]

[1] Structural Biochemistry & Molecular Biophysics Laboratory, Department of Biochemistry, College of Life Science and Biotechnology, Yonsei University, Seoul 03722, Korea; uoonsek1@yonsei.ac.kr (Y.H.); jihye2@spin.yonsei.ac.kr (J.-H.Y.); yejeon@spin.yonsei.ac.kr (Y.-E.J.); jmkim@spin.yonsei.ac.kr (J.K.)
[2] Department of Life Sciences, College of Life Sciences and Biotechnology, Korea University, Seoul 02841, Korea; hsjeong@kbsi.re.kr (H.J.); jaesungwoo@korea.ac.kr (J.-S.W.)
[3] Korea Basic Science Institute, Daejeon 28119, Korea
[4] Department of Systems Biology, College of Life Science and Biotechnology, Yonsei University, Seoul 03722, Korea; think8989@gmail.com (Y.Y.); azzogoory@gmail.com (Y.-j.C.)
[5] PCG-Biotech, Ltd. Yonsei Engineering Research Park, Yonsei University, Seoul 03722, Korea
[6] Research Solution Center, Institute for Basic Science, Daejeon 34126, Korea; ryubh@ibs.re.kr
[7] Department of Chemical and Biological Engineering, College of Engineering, Korea University, Seoul 02841, Korea; lbr0523@korea.ac.kr (B.-R.L.); amyann109@naver.com (S.J.); ww1291@korea.ac.kr (E.J.)
* Correspondence: leejw@korea.ac.kr (J.L.); hscho8@yonsei.ac.kr (H.-S.C.); wlee@spin.yonsei.ac.kr (W.L.)
† These authors contributed equally to this work.

Supplementary Materials

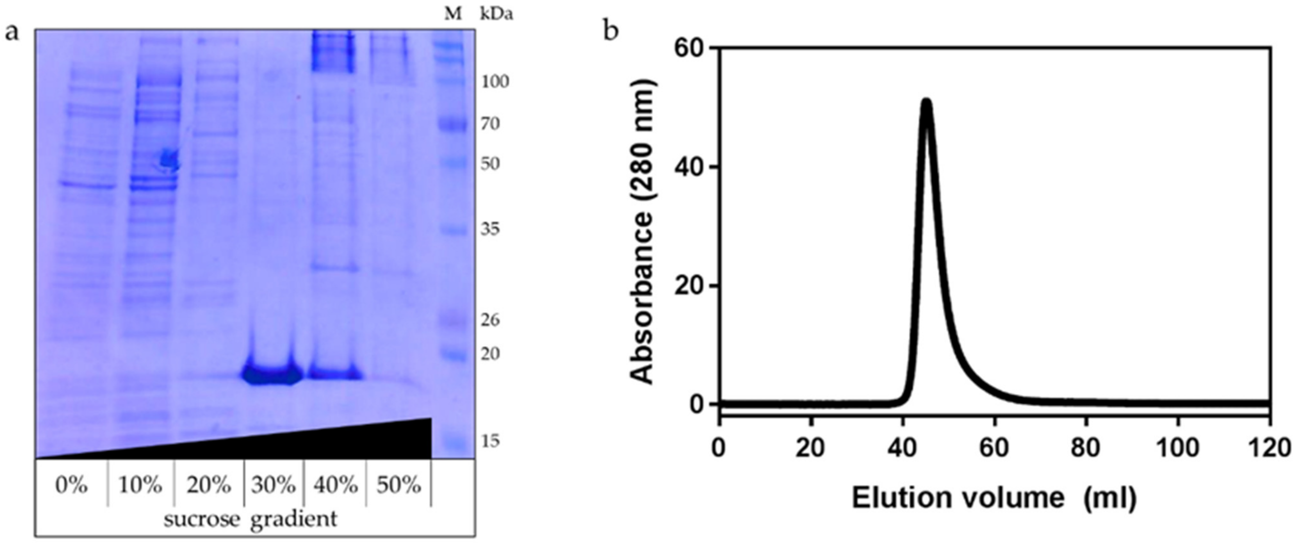

Supplementary Materials Figure S1. Purification of HBcAg. (**a**) Sucrose gradient centrifugation result of HBcAg (MW: 18.99 kDa). The HBcAg was fractionated according to the sucrose concentration (0%–50%). Each fraction was analyzed using SDS-PAGE. The 30% fraction was used for further purification. (**b**) SEC of the finally purified HBcAg. The HBcAg was eluted in void volume.

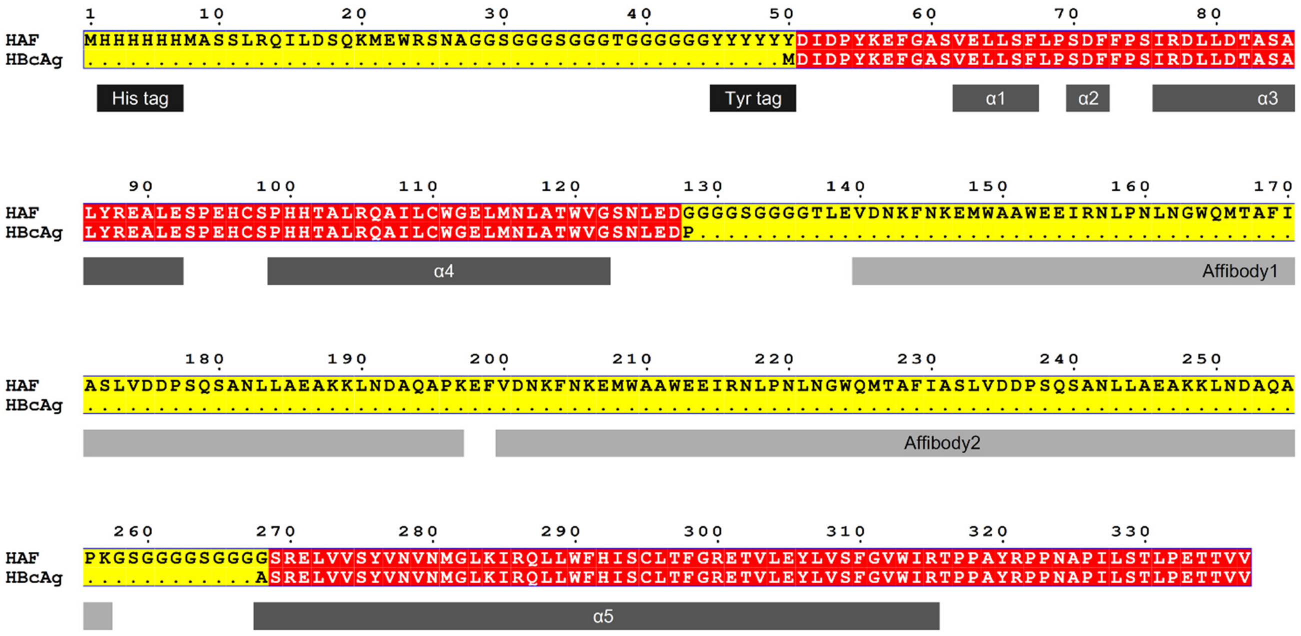

Supplementary Materials Figure S2. Sequence alignment of HAF and HBcAg. Identical residues and non-aligning residues are shown in red and yellow, respectively. The positions of the 6× histidine tag and 6× tyrosine tag are shown as black bars. The dark grey bars and light grey bars reveal the positions of helices and the positions of affibody in the HAF, respectively.

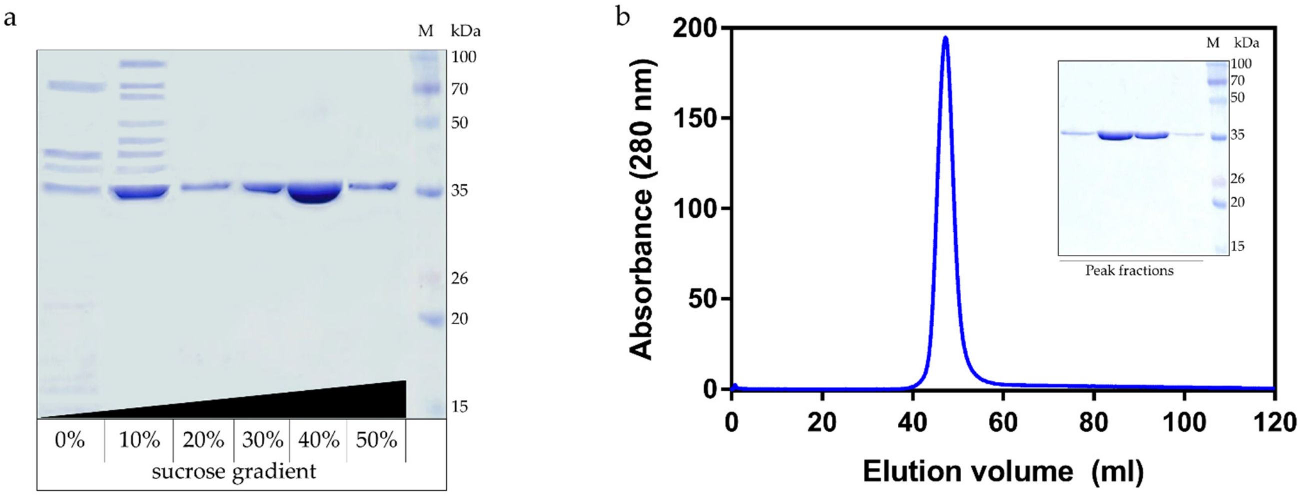

Supplementary Materials Figure S3. Purification of HAF. (**a**) Sucrose gradient centrifugation result of HAF. The HAF was fractionated according to the sucrose concentration (0%–50%). Each fraction was analyzed using SDS-PAGE. The 40% and 50% fractions were used for further purification. (**b**) SEC and SDS-PAGE profile of the finally purified HAF. The HAF was eluted in void volume, and the peak fractions were visualized through SDS-PAGE.

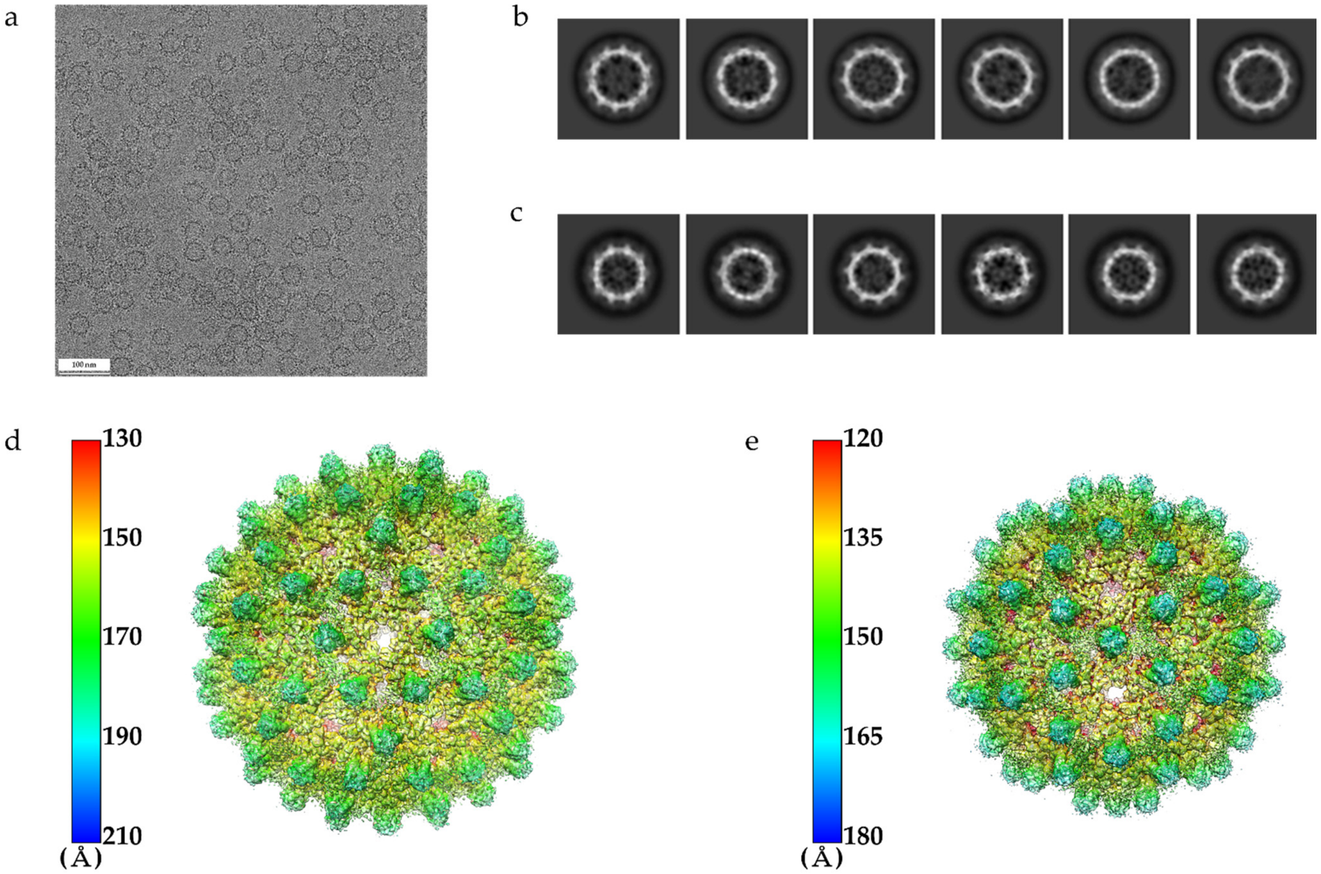

Supplementary Materials Figure S4. The cryo-EM micrograph, 2D class-averaged images, and cryo-EM structures of HAF. (**a**) Cryo-EM micrograph of HAF recorded from Titan Krios 300kV transmission electron microscope. The particles were well dispersed and the number of particles per micrograph was sufficient. (**b**) The most populated 2D class-averaged images of HAF (T=4 state). The image was obtained using 68,577 particles. (**c**) The most populated 2D class-averaged images of HAF (T=3 state). The image was obtained using 50,688 particles. (**d**) The density map and molecular structure of HAF (T=4 state) were superimposed. (**e**) The density map and molecular structure of HAF (T=3 state) were superimposed.

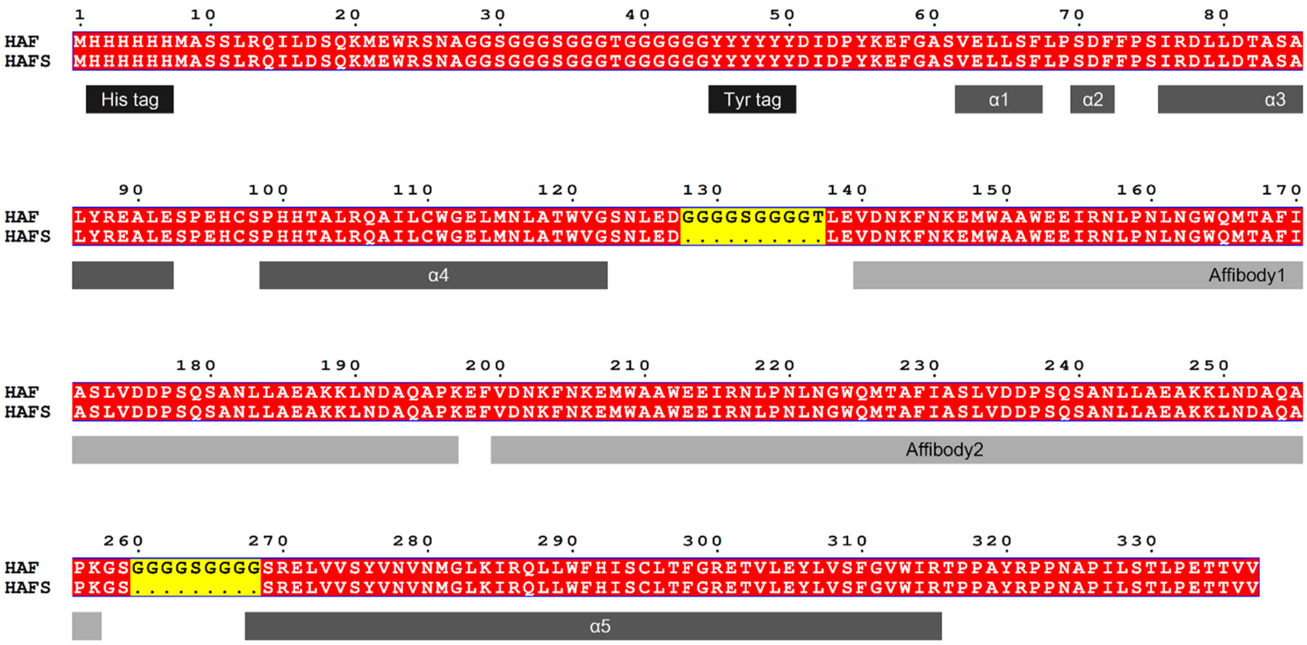

Supplementary Materials Figure S5. Sequence alignment of HAF and HAFS. Identical residues and non-aligning residues are shown in red and yellow, respectively. The positions of the 6× histidine tag and 6× tyrosine tag are shown as black bars. The dark grey bars and light grey bars reveal the positions of helices and the positions of affibody in the HAF and HAFS, respectively.

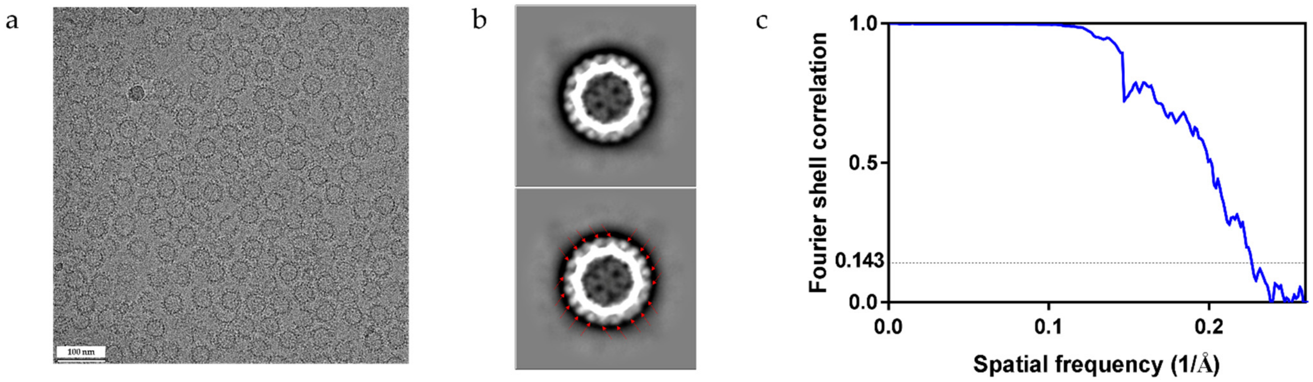

Supplementary Materials Figure S6. The cryo-EM micrograph and 2D class-averaged images of HAFS. (**a**) Cryo-EM micrograph of HAFS recorded from Titan Krios 300kV transmission electron microscope. The particles were well dispersed and the number of particles per micrograph was sufficient. (**b**) The most populated 2D class-averaged image of HAFS (T=4 state). The image was obtained using 46,322 particles. The affibody density is clearly identified in the 2D class-averaged image of HAFS. Each red arrow indicates the observed affibody density. (**c**) FSC curve of the HAFS (T=4 state) reconstruction. The dashed line indicates the correlation of 0.143 which was used as cutoff for determining the resolution. The final resolution is 4.41 Å.

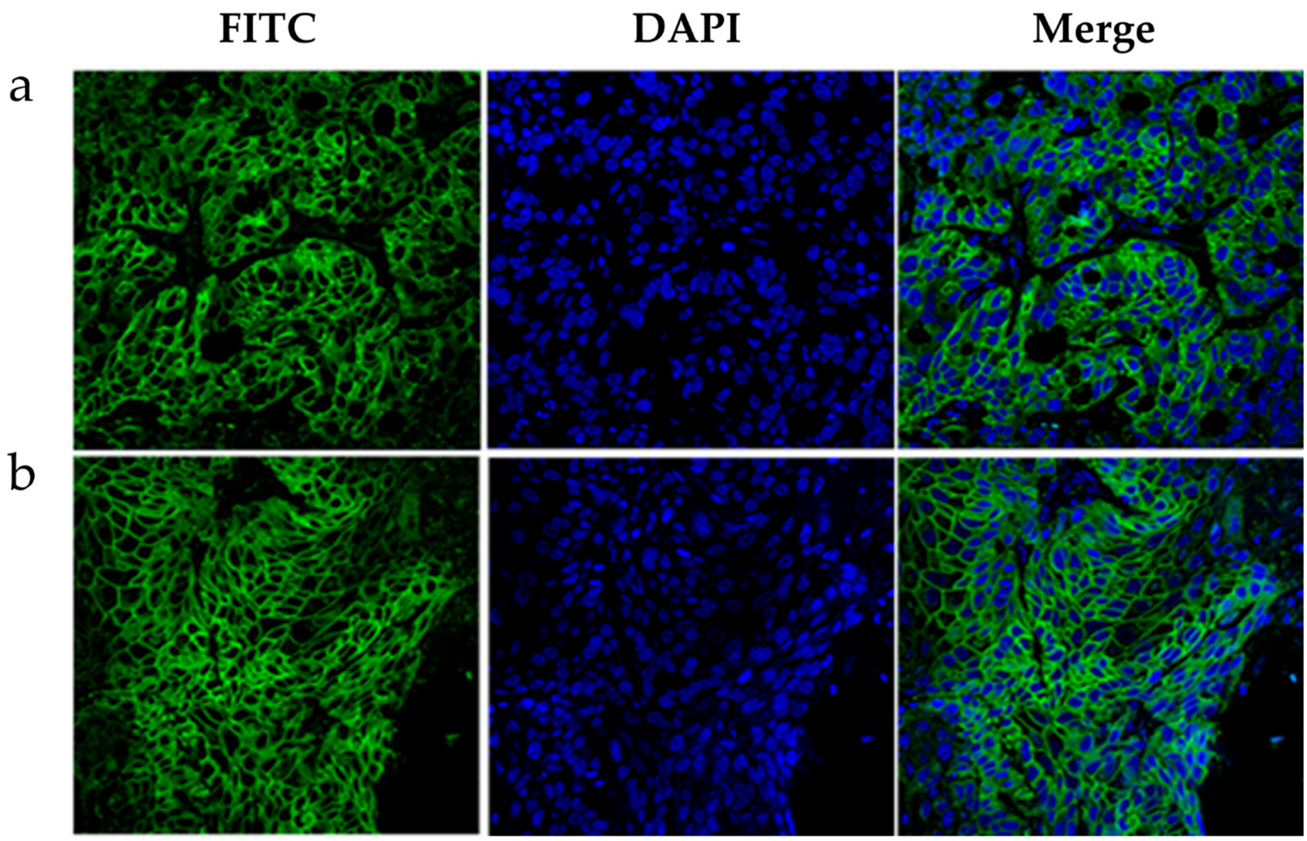

Supplementary Materials Figure S7. EGFR overexpression of HT-29 cells and A431 cells. (**a**) Confocal immunofluorescence images of EGFR overexpressing HT-29 cells. (**b**) Confocal immunofluorescence images of EGFR overexpressing A431 cells. The green and blue signals represent FITC-conjugated IgG to EGFR and DAPI-stained nuclei, respectively.

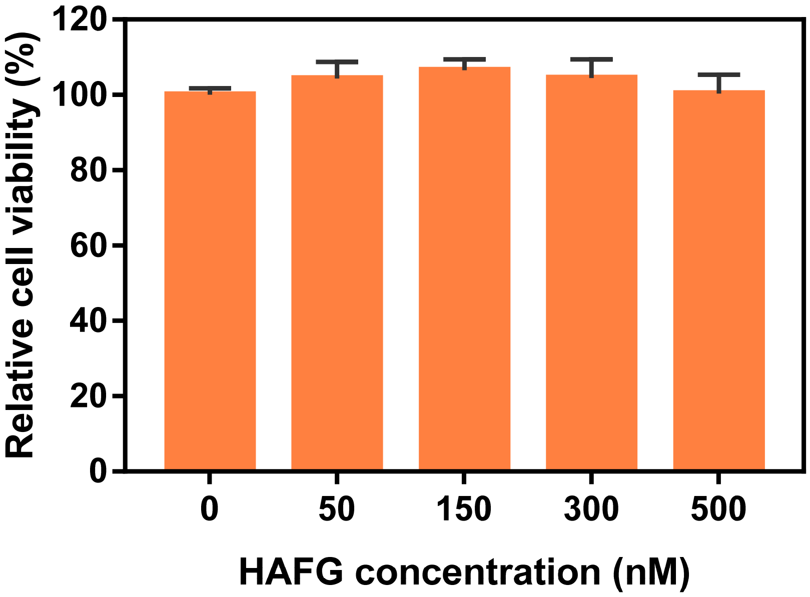

Supplementary Materials Figure S8. The cytotoxicity test of HAFG on WI-38 cells. CCK-8 assay result of human lung fibroblast cell line WI-38. The cell viability was constant regardless of the concentration of HAFG. In this figure, mean and standard deviation are presented (n=6). The viability measured without protein was presented as 100%.
